# Investigation on Mechanical Shock Wave Protective and Thermodynamic Properties of SiO_2_-Aerogel-Modified Polyurea

**DOI:** 10.3390/ma17235817

**Published:** 2024-11-27

**Authors:** Chuanyi Liu, Wenlong Xu, Tonghui Yang, Dong Ma, Shiyu Jia, Zehao Li

**Affiliations:** 1 Institute for Advanced Technology, Shandong University, Jinan 250061, China; 2 School of Energy and Power Engineering, Shandong University, Jinan 250061, China; 3 School of Aerospace Engineering, Tsinghua University, Beijing 100084, China; 4 State Key Laboratory of Explosion Science and Technology, Beijing Institute of Technology, Beijing 100081, China; 5 China Wuzhou Engineering Group Corporation Ltd., Beijing 100053, China

**Keywords:** polyurea, SiO_2_ aerogel, mechanical shock wave mitigation, thermodynamics properties

## Abstract

In recent years, industrial explosion accidents are frequent, causing serious negative influences on society. Mechanical shock waves, as a typical destructive factor in explosion accidents, can cause serious personal injury and building damage. In addition, actual explosion accidents usually involve heat sources, harming protective materials and personnel. In this study, we designed SiO_2_-aerogel-modified polyurea and studied the effects of manufacturing pressure process and the concentration of SiO_2_ aerogel on the mechanical shock wave mitigation and thermodynamic properties of the modified polyurea. The results show that the addition of SiO_2_ aerogel can improve the mechanical shock wave mitigation performance of polyurea. The maximum peak overpressure and acceleration mitigation rate of the material has reached 17.84% and 62.21%, respectively. The addition of SiO_2_ aerogel helps to reduce the thermal conductivity of materials and improve the thermal insulation performance, and the atmospheric pressure process is more conducive to improving the thermal insulation performance of materials. The minimum thermal conductivity of the material has reached 0.14174 W/m·K, which is 45.65% lower than that of pure polyurea. The addition of SiO_2_ aerogel has different effects on the limiting oxygen index (LOI) of polyurea. Using a vacuum process, the LOI value increased with the increase in the SiO_2_ aerogel concentration, while using atmospheric pressure, the LOI value increased but is always lower than 21% and lower than pure polyurea. Thermogravimetric analysis showed that the addition of SiO_2_ aerogel under the vacuum process was helpful to improve the thermal stability of materials. However, atmospheric pressure would disrupt the thermal stability, manifested in a decrease in peak degradation temperature, an increase in peak degradation rate, and a decrease in residual mass.

## 1. Introduction

In recent years, industrial explosion accidents have occurred frequently, with great destructive power, leading to negative impacts on social order and economic development. Mechanical shock waves are a typical destructive factor. A mechanical shock wave is a high-speed, high-pressure transient air wave formed by the release of enormous energy from explosions. Mechanical shock waves can cause serious physical injuries [1,2,3,4], such as eardrum perforation, lung contusion, traumatic brain injury (TBI), multi-organ laceration and bleeding, severe disability, and even death. Mechanical shock waves can also cause damage to surrounding buildings and structures, leading to glass breakage, building collapse, etc. In addition, there are always some heat sources in actual explosion accidents, and the thermal radiation of these heat sources has the characteristics of high radiation temperature, wide radiation area, and long duration. They usually have strong penetrability, causing thermal decomposition failure of protective materials and personnel burns. It can be seen that it is urgent and necessary to study the mechanical shock wave protection and thermodynamic properties of safety engineering protective materials.

Polyurea [5,6,7,8] is a polymer copolymer with good flexibility, mechanical strength comparable to metal materials, light weight, composability with other materials, and easy processing, which has attracted widespread attention from researchers. Song et al. [9] conducted contact explosion tests on straight reinforced-concrete walls and found that the surface damage area depth of C50 concrete coated with five layers of polyurea was reduced by 38% compared to C70 concrete without polyurea coating after the explosion. Zhu et al. [10] found that under the repeated explosions, compared to aluminum alloy plates without polyurea coating, plates coated with polyurea on the back increased the local and overall deformation resistance by 33.9% and 31.3%, respectively, while plates coated with polyurea on both sides increased the resistance by 19.3% and 22.5%, respectively. Jia et al. [11] studied shock wave protection performance of the structure with polyurea placed at the multi-layer flexible composite. The thickness of polyurea and polyurethane foam was 1.0 mm and 4.0 mm, respectively, and the overpressure attenuation rate reached the maximum value of 93.3%, which was better than the single-layer polyurethane foam with high surface density. These studies have demonstrated the great ability of polyurea to mitigate mechanical shock waves. However, these studies mainly focus on the performance of polyurea in combination with other materials, lacking research on the inherent properties of polyurea mechanical shock wave mitigation. In addition, there is relatively little research on the thermodynamic properties of polyurea materials.

Researchers have conducted much work to improve the mechanical shock wave protection performance of materials, and a common strategy is to design structures to dissipate the blast energy. According to structural scales, there are generally some types of materials, such as multi-layer composite materials [12,13,14,15], filler inclusion materials [16,17,18,19,20,21,22,23,24,25], and dynamic molecular bonds introduced into polymer matrix [26,27]. Among them, the filler modification material process can change the intrinsic properties of the material, and it is low-cost and easy to operate. The mechanical shock wave mitigation of heterogeneous polymer composites with added fillers can be attributed to various mechanisms, such as filler-induced stress concentration [19], viscosity-induced dissipation [20,21], scattering of shock wave energy by fillers [16], interfacial phonon-damping effect [17], and the mitigation properties of the fillers themselves [18], etc. In addition, adding fillers to improve the shock wave mitigation properties of materials is influenced by various factors, such as the type, size, concentration, dispersion of the fillers in the polymer matrix, and the interaction between the microstructure of the filler surface and the polymer matrix. Rauls and Ravichandran [25] embedded silica glass spheres in thermoplastic polymer matrix and studied the shock wave structure through experiments and numerical simulations. The research shows that the appropriate size and uniform distribution of the filler particles can effectively increase the rise time of the shock wave, optimizing the shock wave structure. Moumen et al. [22] conducted a shock wave mitigation test on epoxy polymers with different concentrations of CNTs (1 wt%, 2 wt%, and 4 wt%). It was found that the shock wave intensity was reduced by 33.34% for 4% CNTs compared to 0% CNTs. Wei et al. [24] studied PDMS composite materials with particle-mediated structures and reported that compared with pure PDMS, the shock wave peak pressure for composites with agglomerate structure and homodisperse structure reduced by up to 43% and 75%, respectively. An et al. [23] fabricated a polymer composite by complexation of polyacrylic acid, polyvinylpyrrolidone, and carbonized polymer dots (CPDs). The functional groups on the CPDs filler surface form strong non-covalent interactions with the polymer matrix, effectively transferring and dissipating impact energy.

SiO_2_ aerogel is a kind of high-performance aerogel material [28,29,30,31], which has extremely low density, high specific surface area, extremely low thermal conductivity, high temperature resistance, and other properties. Typical SiO_2_ aerogels [32,33,34] contain primary particles with a diameter of 2–5 nm, which are assembled into a structure similar to a pearl necklace, and eventually form secondary particles with a diameter of 50–100 nm, whose size is similar to that of common effective nano fillers. Its internal particles are composed of SiO_2_, which is almost completely polymerized, and its surface is covered with hydroxyl (-OH) group, which can react with isocyanate group, one of the polyurea raw materials, to realize the covalent crosslinking between particles filler and polyurea matrix. The water molecule of SiO_2_ aerogel will react with the polyurea matrix to produce CO_2_ bubbles. The bubbles left in the matrix will form holes, which may refract and reflect the mechanical shock wave, affecting the mechanical shock wave mitigation performance of the material. The generation of holes in the matrix can be controlled by controlling the vacuum process and atmospheric pressure process during the preparation process.

In this study, SiO_2_-aerogel-modified polyurea was designed. The effects of the vacuum process and the concentration of SiO_2_ aerogel on the mechanical shock mitigation and thermodynamic properties of modified polyurea materials were studied. Through controlling the vacuum pump during the manufacturing process, the vacuum process (6 × 10^−2^ Pa) and the atmospheric pressure process (1.01 × 10^5^ Pa) were realized. The mechanical shock wave mitigation performance of the material was evaluated through mechanical shock wave experiments. The experiment was conducted on the mechanical shock wave experimental system, which includes a multi-target shockwave cannon (MSC) and a human tissue equivalent target (HTET). To evaluate the mechanical shock wave mitigation performance of materials, two baseline models were established, which improved the robustness of the mechanical shock wave experimental system. The thermodynamic properties were tested by thermal conductivity experiments, limiting oxygen index (LOI) experiments, and thermogravimetric (TG) experiments. Thermal conductivity was used to evaluate the thermal insulation performance of materials, LOI was used to evaluate the flame retardant performance of materials, and TG experiments were used to evaluate the thermal stability of materials. In the Section 2, the detailed preparation process of SiO_2_-aerogel-modified polyurea was introduced. In the Section 3, specific experimental methods for mechanical shock wave mitigation performance and thermodynamic properties of materials were introduced. In the Section 4, the results obtained from experiments were shown and discussed. In the Section 5, the final conclusion was reached.

## 2. Materials and Methods

In this experiment, the polyurea matrix was synthesized from 30% carbodiimide modified diphenylmethane diisocyanate (CDMDI) (component A) and 70% poly-1,4-butanediol bis (4-aminobenzoate) (component B). The SiO_2_ aerogel fillers were produced by Fanruiyihui Composite Material Co., Ltd. (Zhengzhou, China). The microparticle size of the SiO_2_ aerogel fillers is 20–60 nm, with a specific surface area of 500–1000 m^2^/g and a tap density of 40–60 kg/m^3^. In this work, two types of SiO_2_-aerogel-modified polyurea were prepared by distinct vacuum process and SiO_2_ aerogel mass concentration during the manufacturing process. Through controlling the vacuum pump during the manufacturing process, the vacuum process (6 × 10^−2^ Pa) and the atmospheric pressure process (1.01 × 10^5^ Pa) were realized.

**Figure 1:**
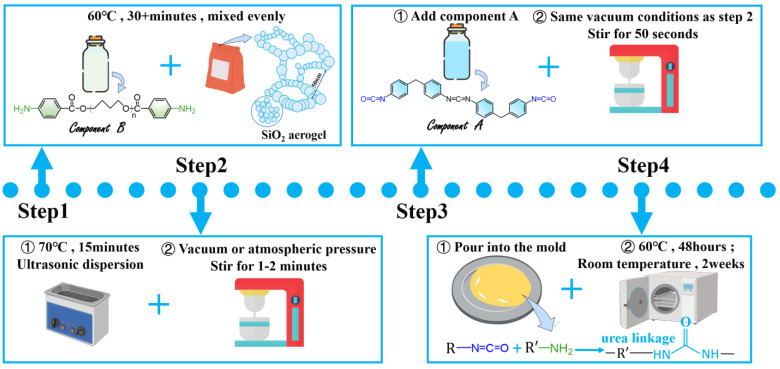
Material preparation process.

The preparation method is shown in Figure 1, and the SiO_2_ aerogel mass concentration and process are shown in Table 1. The preparation process can be divided into 4 steps. Step 1: Add SiO_2_ aerogel of a certain quality to 70% component B, stir them evenly, place them at 60 °C for more than 30 min, and make them fully heated and melted, with good fluidity and low viscosity. Step 2: Place the mixture into an ultrasonic disperser for ultrasonic dispersion. Set the temperature to 70 °C, and time to 15 min. After dispersion is complete, stir the mixture thoroughly for 1–2 min under vacuum (6 × 10^−2^ Pa) or atmospheric pressure (1.01 × 10^5^ Pa) conditions, and stir evenly. Step 3: Add 30% component A under the same vacuum conditions and stir for 50 s to mix evenly. Step 4: Quickly pour it into the mold, place it in a 60 °C constant temperature drying oven, keep it at 60 °C for 48 h. Then, take it out, and let it stand at room temperature for two weeks. Finally, obtain the material.

The materials were named according to the mass concentration of SiO_2_ aerogel and the vacuum conditions. Pure polyurea was represented as PU. As shown in Figure 1, the polyurea modified by SiO_2_ aerogel was expressed as PA. The number after the letter PA indicated the mass concentration of SiO_2_ aerogel in polyurea matrix. According to the different processes used in the production process, the suffix of vacuum process (6 × 10^−2^ Pa) materials was V, and the suffix of atmospheric pressure (1.01 × 10^5^ Pa) process materials was N.

## 3. Experimental Methods

### 3.1. Microscopic Morphology Observation

The microstructure of the polyurea composites was observed with scanning electron microscopy (SEM), using equipment model JSM-7610F, produced by Japan Electronics Corporation (Tokyo, Japan). Energy-dispersive spectroscopy (EDS) was used to analyze the types and distributions of elements in the micro regions of the material and then to analyze the existence form of SiO_2_ aerogel fillers in the material. The equipment model is X-max, produced by Oxford, UK.

### 3.2. Mechanical Shock Wave Experiments and Evaluation Methods

#### 3.2.1. Mechanical Shock Wave Experiments

**Figure 2:**
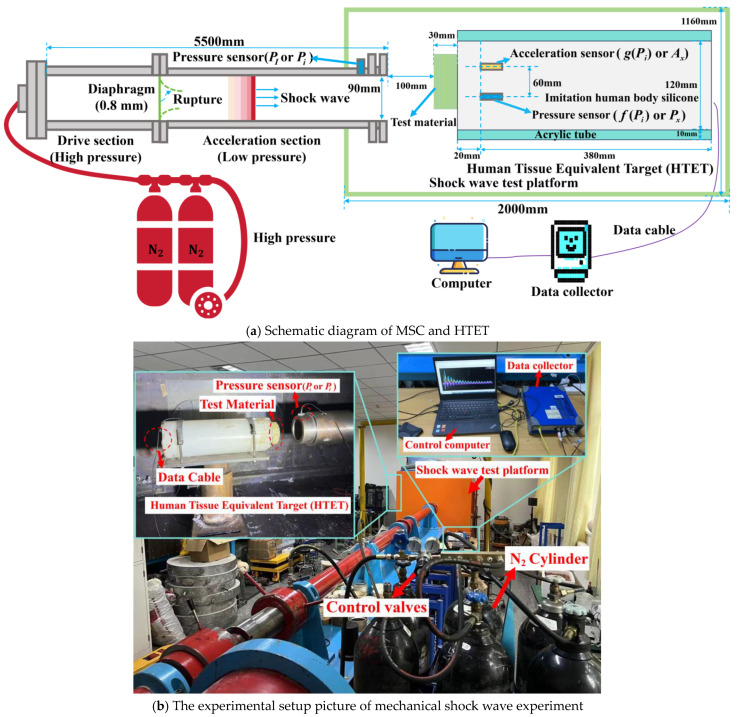
Mechanical shock wave experimental setup.

The schematic diagram of the experimental setup is shown in Figure 2a, and the actual experimental setup is shown in Figure 2b. The experiment was conducted on the “Multi-purpose Shock Cannon” (MSC) at the Transient Physics Laboratory of Shandong University (Weihai, China). As shown in Figure 2a, the device was independently developed by the team and can generate high-pressure and high-speed shock wave airflow for simulating mechanical shock waves generated by explosions. This device is safer, easier to operate, and can generate more stable mechanical shock wave pressure than traditional explosion loading systems. The device includes a 5.5-meter-long shock tube, which is divided into two sections: the drive section and the acceleration section, with a 0.8 mm thick aluminum diaphragm placed between them. Injecting 3 Mpa of nitrogen gas into the driving section until the diaphragm ruptures, the gas accelerated and was ejected through the acceleration section, forming a mechanical shock wave. A pressure sensor (model: Kistler 603CBA000020.0, range: 200 bar, sensitivity: 27.62 mv/bar, Winterthur, Switzerland) was installed at muzzle, capable of recording air pressure to characterize the intensity of mechanical shock waves.

In order to simulate the response of human tissue to mechanical shock waves, the team developed a human tissue equivalent target (HTET). HTET was cylindrical in shape, filled with silicone medium inside, and its mechanical properties were similar to real human tissue. A pressure sensor (model: Kistler 603CBA000070.0, measurement range: 88.9 bar, sensitivity: 70.6 mv/bar) and an acceleration sensor (model: Kistler 8742A5, range: 5000.00 g, sensitivity: 1.01 mv/g) were installed at a distance of 2 cm from HTET end face to collect stress wave signals.

The data collected by the sensor were processed by a charge amplifier and recorded by a data collection instrument to obtain real-time pressure and acceleration inside the HTET. The modified polyurea material specimens were placed in front of the HTET. These materials were cut into circular shapes with a diameter of 90 mm, stacked in 6 layers, with a total thickness of about 3 cm, and made into specimens. Through MSC, the mechanical shock wave was released to the HTET where the specimen was placed. Muzzle pressure signals, pressure, and acceleration signals inside the HTET were collected. The mechanical shock wave mitigation performance of the material was assessed based on the peak values of these signals.

#### 3.2.2. Evaluation Methods and Indicators

##### Baseline Models

**Figure 3:**
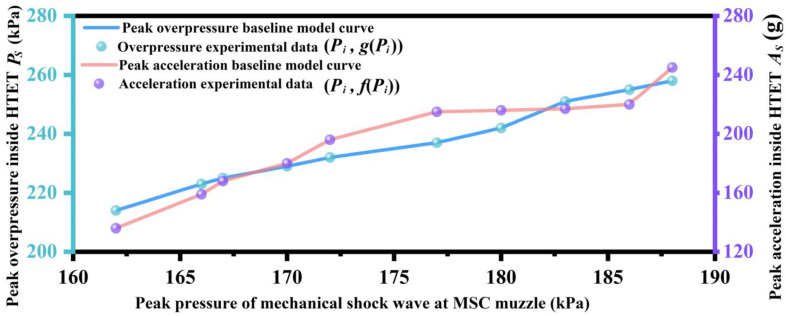
Peak overpressure baseline model and peak acceleration baseline model.

Due to factors such as the precision of diaphragm processing, the pressure of gas inside the cylinder, and the temperature of the experimental environment, slight random fluctuations in muzzle pressure are normal. Before testing the mechanical shock wave mitigation performance of the materials, two baseline model curves were established: peak overpressure baseline model and peak acceleration baseline model. The peak overpressure baseline model was used to describe the relationship between the peak pressure of mechanical shock wave at MSC muzzle and the peak overpressure inside HTET. The peak acceleration baseline model was used to describe the relationship between the peak pressure of mechanical shock wave at MSC muzzle and the peak acceleration inside HTET. Without placing any samples, MSC was used to repeatedly release mechanical shock waves to HTET under the same operating conditions, and a total of 10 sets of data were collected. Each set of data includes three physical parameters: the peak pressure of mechanical shock wave at MSC muzzle, the peak overpressure, and the peak acceleration inside the HTET. Through programming Lagrange interpolation using a computer, two baseline model curves were accurately calculated using these 10 sets of data. The model curves can essentially be considered as the result of Taylor series expansion of the original function within a specific interval, as shown in Figure 3. The specific calculation process is as follows:

(1) $$P_{S}\left(P_{I}\right)=\sum_{i=1}^{n+1}f\left(P_{i}\right)\left(\prod_{j\neq i}^{1\leq j\leq n}\frac{\left(P_{I}-P_{i}\right)}{\left(P_{i}-P_{j}\right)}\right)$$

(2) $$A_{S}\left(P_{I}\right)=\sum_{i=1}^{n+1}g\left(P_{i}\right)\left(\prod_{j\neq i}^{1\leq j\leq n}\frac{\left(P_{I}-P_{i}\right)}{\left(P_{i}-P_{j}\right)}\right)$$

where *P_I_* is the measured peak pressure of mechanical shock wave at MSC muzzle in each experiment. *P_S_* is the theoretical peak overpressure inside HTET, and *A_S_* is the theoretical peak acceleration inside HTET, which were all calculated by the Lagrange interpolation. *P_i_*, *f*(*P_i_*), *g*(*P_i_*) (*i* = 0, 1, 2, …, *n*−1; *n* = 10) are the 10 sets of experimental data obtained by MSC and HTET without placing samples under the same working conditions. *P_i_* is the peak pressure of the mechanical shock wave at MSC muzzle, *f*(*P_i_*) is the measured peak overpressure inside HTET, and *g*(*P_i_*) is the measured peak acceleration inside HTET.

##### Evaluation Indicators

To assess the mechanical shock wave mitigation performance of different materials, two rates are employed for detailed analysis: peak overpressure mitigation rate (γ*_p_*) and peak acceleration mitigation rate (γ*_a_*). The mitigation rates were calculated by two physical parameters: peak overpressure and peak acceleration. Comparing parameters of HTET protected by modified polyurea with parameters of HTET without any protective material, the calculation process is as follows:

(3) $$\gamma_{p}=\frac{P_{S}-P_{x}}{P_{S}}$$

(4) $$\gamma_{a}=\frac{A_{S}-A_{x}}{A_{S}}$$

where *P_S_* and *A_S_* are theoretical peak overpressure and peak acceleration of HTET without protective material, and they are calculated by the baseline models. *P_x_* and *A_x_* are the measured peak overpressure and peak acceleration of HTET protected by modified polyurea composites in each experiment, respectively. The mitigation rates of each sample were calculated by program operation.

### 3.3. Thermodynamic Properties Experiments

#### 3.3.1. Thermal Conductivity Experiments

The thermal insulation effect of the materials was evaluated based on their thermal conductivity. Generally speaking, materials with lower thermal conductivity have better thermal insulation effects. In this study, the method for testing thermal conductivity was the transient plane heat source method, and the experimental instrument was the DRE-III multifunctional rapid thermal conductivity tester produced by Xiangtan Xiangyi Instrument Co., Ltd. (Changsha, China). Two samples of the same specifications were made for each material for testing.

#### 3.3.2. Limiting Oxygen Index Experiments

Limiting Oxygen Index (LOI) is usually used for evaluating the fire retardancy of materials. A high LOI indicates that the material has good fire retardancy properties. An oxygen index tester (model: JF-3, Beijing, China) was employed for LOI testing. The test sample is processed into a rectangular prism of 8 × 80 × 4 mm and tested for the minimum oxygen concentration required to maintain continuous combustion. This concentration is LOI value of the sample.

#### 3.3.3. Thermogravimetric Experiments

The thermal stability of the sample was evaluated through thermogravimetric (TG) experiments. Thermogravimetric (TG) analysis and differential thermogravimetric analysis (DTG) graphs were plotted to describe the experimental results. The reference parameters were residual mass fraction displayed in the TG graph, and the peak degradation rate and peak degradation temperature displayed in the DTG graph. The experiment was conducted on a thermogravimetric analyzer (model NETZSCH TG 209F3, Selb, Germany) in a nitrogen atmosphere, with a temperature range of 0–800 °C and a heating rate of 20 °C/min.

## 4. Results and Analysis

### 4.1. Microscopic Morphology Analysis

**Figure 4:**
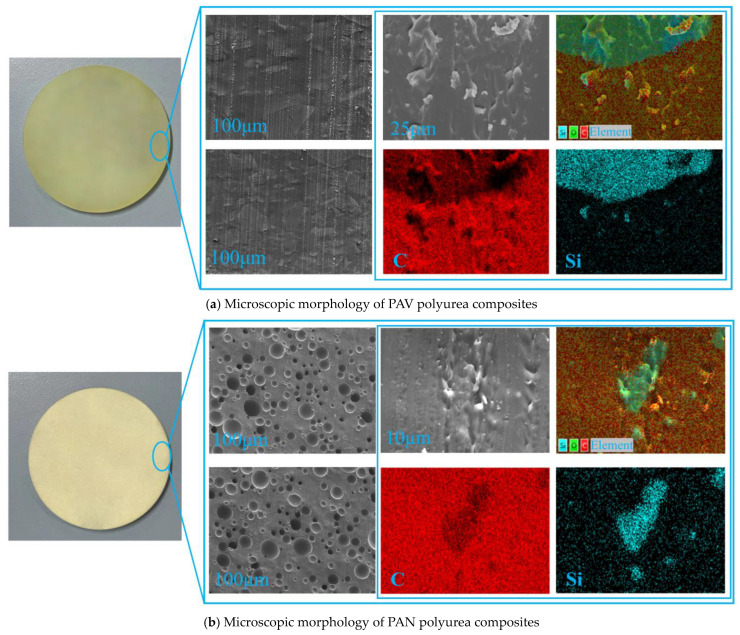
Microscopic morphology of SiO_2_-aerogel-modified polyurea composites.

The microstructure of the material was observed through SEM. As shown in Figure 4, there was a significant difference in morphology between PAN and PAV polyurea composites. As shown in Figure 4a, the surface of PAV polyurea composites was relatively flat, while there were many holes with diameters of tens of micrometers inside PAN polyurea composites, as shown in Figure 4b. It was obvious that this was due to the difference between vacuum process and atmospheric pressure process. The reason was that the water molecule of the SiO_2_ aerogel reacts with component A to produce CO_2_ gas. The gas was extracted by the vacuum process adopted by PAV polyurea composites, so that the matrix was even and free of holes. The atmospheric pressure process adopted by PAN polyurea composites leaves the bubbles in the matrix, forming holes. Holes would refract and reflect mechanical shock waves, promoting the dissipation of mechanical shock wave energy.

The morphology of SiO_2_ aerogel fillers in the matrix was analyzed through EDS. As shown in Figure 4, SiO_2_ aerogel could be marked with its unique Si element, while the polyurea matrix could be marked with its unique C element, and related substances can be represented by characteristic elements. As shown in Figure 4, at the scale of 25 μm, the SiO_2_ aerogels were clustered and did not form a unified whole with the polyurea matrix. In the bright micro region of the Si element, the C element was dark, indicating that the SiO_2_ aerogel was mixed in the matrix and was not completely dissolved by the matrix.

### 4.2. Mechanical Shock Wave Experiments Results and Analysis

#### 4.2.1. Peak Overpressure Mitigation Analysis

**Figure 5:**
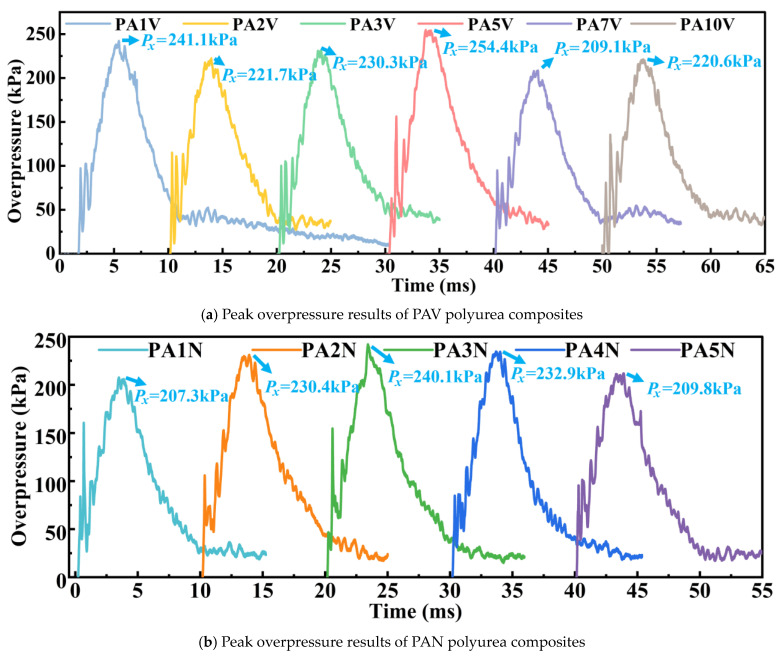
Mechanical shock wave experiment overpressure results.

**Figure 6:**
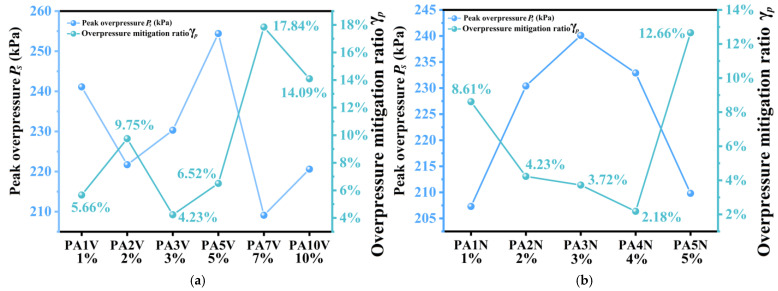
Overpressure mitigation trend of polyurea composites with different filler concentrations. (**a**) Overpressure mitigation trend rates of PAV polyurea composites. (**b**) Overpressure mitigation trend rates of PAN polyurea composites.

The overpressure experiments results of PAV polyurea composites are shown in Figure 5a, and the overpressure experiments results of PAN materials are shown in Figure 5b. Due to slight fluctuations in muzzle pressure, the measured peak overpressure of the material may randomly increase or decrease. Therefore, the raw data of the experiment need to be converted through a unified standard to compare and analyze the mitigation performance of each material, calculate the peak overpressure mitigation rate, and this unified standard is the baseline model. As shown in Figure 6a, with increasing concentrations of the SiO_2_ aerogel, the γ*_p_* values of the PAV polyurea composites first decreased and then went up, with an average of 8.48%. The “average” here refers to the sum of the γ*_p_* values of a material divided by the number of material types. The γ*_p_* value of PA7V reached 17.84%, showing great peak overpressure mitigation performance. As shown in Figure 6b, for PAN polyurea composites, with the increase in filler concentration, γ*_p_* values declined first and rose later, with an average of 6.28%. PA5N polyurea, with γ*_p_* value of 12.66%, had the best mitigation performance in PAN type polyurea materials, which was better than PA5V polyurea. This showed that using this formula, the atmospheric pressure process was conducive to the mechanical shock wave mitigation performance of the material. A possible reason was that the pores inside the material could refract and reflect the wave, promoting energy dissipation.

#### 4.2.2. Peak Acceleration Mitigation Analysis

**Figure 7:**
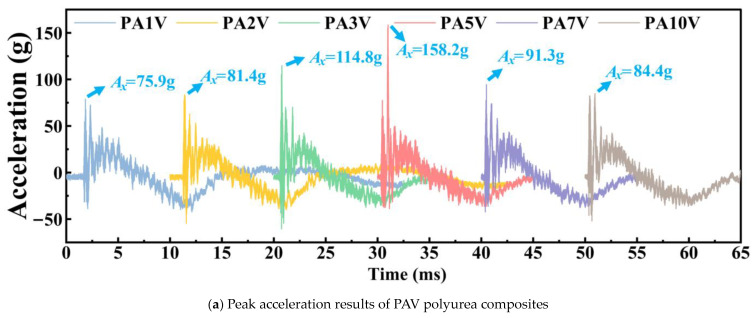

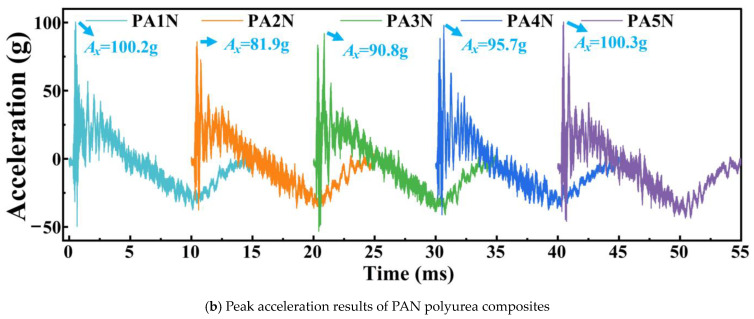
Mechanical shock wave experiment acceleration results.

**Figure 8:**
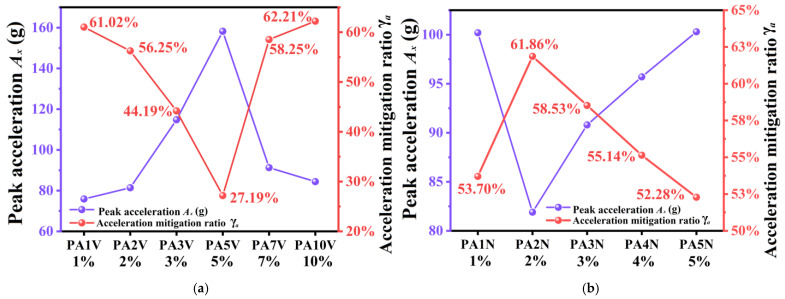
Overpressure mitigation trend of polyurea with different filler concentration. (**a**) Overpressure mitigation trend rates of PAV polyurea composites. (**b**) Overpressure mitigation trend rates of PAN polyurea composites.

The acceleration experiments results of PAV polyurea composites are shown in Figure 7a, and the acceleration experiments results of PAN polyurea composites are shown in Figure 7b. As shown in Figure 8a, with increasing concentrations of the SiO_2_ aerogel, the γ*_a_* values of PAV polyurea composites first declined and then increased, with an average of 51.52%.The γ*_a_* values of PA1V and PA10V reached 61.02% and 62.21%, respectively. PA5V had the worst mitigation performance on acceleration peak, with a γ*_a_* value of 27.19%. This showed that a low or high concentration of the SiO_2_ aerogel was beneficial to the polyurea composites mitigation performance of shock wave acceleration, which may be related to the filler existence form in the matrix. As shown in Figure 8b, with increasing concentrations of the SiO_2_ aerogel, the γ*_a_* values of the PAN polyurea composites first went up and then decreased, with an average of 56.30%. Among them, PA1N and PA5N polyurea had the worst performance, with mitigation of about 53.70% and 52.28%, respectively. PA2N polyurea had the best mitigation performance on acceleration peak, with a value of 61.86%. The data showed that when adding the SiO_2_ aerogel, using the atmospheric pressure process was conducive to polyurea shock wave energy dissipation.

### 4.3. Thermodynamic Properties Experiments Results and Analysis

#### 4.3.1. Thermal Conductivity Experiments Results and Analysis

**Figure 9:**
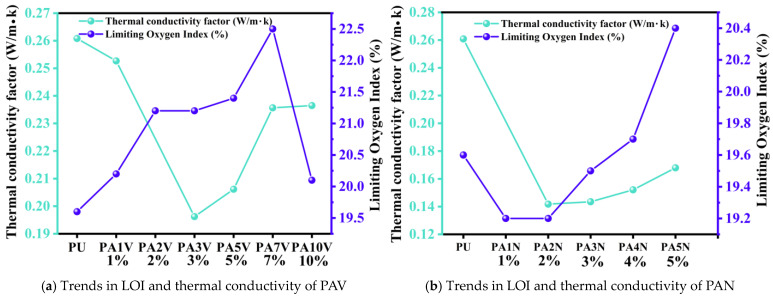
Trend in LOI and thermal conductivity of polyurea composites with different filler concentration.

The detailed data were shown in Table 2. For PAV type polyurea, the vacuum process was used during the manufacturing process. The overall thermal conductivity values ranged from 0.19 W/m·K to 0.25 W/m·K. As shown in Figure 9a, its thermal insulation performance first rose and then decreased with the increasing concentration of SiO_2_ aerogel. The thermal conductivity of PA3V reached 0.19621 W/m·K, which was the lowest thermal conductivity and had the best thermal insulation performance among the PAV polyurea composites, a thermal conductivity decrease of 24.8% compared to PU. For the PAN polyurea composites, the atmospheric pressure process was used during the manufacturing process. The overall thermal conductivity values ranged from 0.14 W/m·K to 0.17 W/m·K. As shown in Figure 9b, its thermal insulation performance first increased rapidly and then decreased slightly with the increasing concentration of the SiO_2_ aerogel. The thermal conductivity of PA2N reached 0.14174 W/m·K, which was the lowest thermal conductivity and had the best thermal insulation performance among the PAN polyurea composites, a thermal conductivity decrease of 45.65% compared to PU. The thermal insulation performance of the PAN polyurea composites was better than that of all PAV-type polyurea, due to the micron-scale pores within the matrix, indicating that the atmospheric pressure process in production will improve the thermal insulation performance of the material.

#### 4.3.2. Limiting Oxygen Index Experiments Results and Analysis

The LOI of all materials is shown in Table 2, and the effect of adding SiO_2_ aerogel on the LOI of the material is shown in Figure 9. The oxygen content in air was about 21%. The LOI of the PU material was 19.6%, which was very flammable in the air environment. For the PAV polyurea composites, as shown in Figure 9a, the LOI first rose and then decreased with the increasing concentration of SiO_2_ aerogel. The LOI of PA2V, PA3V, PA5V, and PA7V was higher than 21%, indicating that the appropriate concentration addition of SiO_2_ aerogel helped to improve the flame retardancy of the material under the vacuum process. For the PAN polyurea, as shown in Figure 9b, the LOI increased with the increasing concentration of SiO_2_ aerogel, but the LOI was lower than 21% in all cases, making them flammable. The LOI values of these materials were lower than those of pure polyurea. This indicated that the atmospheric pressure process in production would reduce the flame retardancy of the material.

#### 4.3.3. Thermogravimetric Experiments Results and Analysis

**Figure 10:**
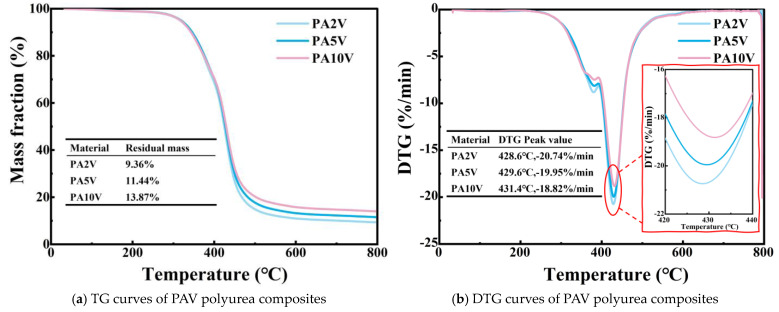

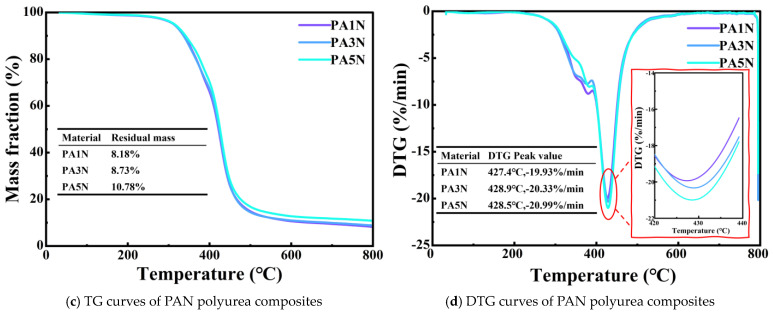
Thermogravimetric experiments results.

The thermogravimetric experiments results of the PAV type material were analyzed, and it was found that the addition of the SiO_2_ aerogel helped to improve the thermal stability of the material. For the PA2V, PA5V, and PA10V polyurea composites, as shown in Figure 10a, the residual mass increased with the increase in the SiO_2_ aerogel concentration, indicating that the presence of SiO_2_ aerogel was conducive to improving residual mass. For the peak degradation temperature, as shown in Figure 10b, PA10V was 431.4 °C, 1.8 °C higher than PA5V and 2.8 °C higher than PA2V, indicating that the increasing concentration of SiO_2_ aerogel helped to increase the peak degradation temperature. For the peak degradation rate, as shown in Figure 10b, PA2V was −20.74%/min, PA5V was −19.95%/min, and PA10V was −18.82%/min, which indicated that the increasing concentration of SiO_2_ aerogel helped to reduce the peak degradation rate of the material. These results showed that under the vacuum pressure process, the addition of the SiO_2_ aerogel enhanced the thermal stability of polyurea.

The thermogravimetric experiments results of the PAN polyurea composites were analyzed, and it was found that compared with PAV, the atmospheric pressure process could reduce the thermal stability of the material. As shown in Figure 10c, the residual mass also increased with the increase in the SiO_2_ aerogel concentration, indicating that the presence of SiO_2_ aerogel was conducive to improving residual mass. As shown in Figure 10d, the peak degradation temperature values ranged from 427.4 °C to 428.5 °C, which were lower than that of PA2V; even the concentration of SiO_2_ aerogel was higher than PA2V. For the peak degradation rate, as shown in Figure 10d, PA5N was −20.99%/min, PA3N was −20.33%/min, and PA1N was −19.93%/min, which indicated that when using the atmospheric pressure process, the increasing concentration of the SiO_2_ aerogel increased the peak degradation rate of the material. These results showed that under the atmospheric pressure process, the addition of the SiO_2_ aerogel broke the thermal stability of the polyurea.

## 5. Conclusions

In this study, SiO_2_-aerogel-modified polyurea composites were prepared. To be specific, under the vacuum process and the atmospheric pressure process, we prepared polyurea composites with different concentrations of SiO_2_ aerogel. The mechanical shock wave protection and thermodynamic properties of different materials were studied through mechanical shock wave mitigation experiments, thermal conductivity experiments, LOI experiments, and TG experiments. The main conclusions are as follows:

1. The mechanical shock wave experiment explored the shock wave mitigation performance of polyurea composites. For polyurea composites using the vacuum process, γ*_p_* values first decrease and then go up with the increase in SiO_2_ aerogel concentration, and the change trend of γ*_a_* values is similar to that of γ*_p_*. The polyurea composites with the best mitigation performance is PA7V, with a γ*_p_* of 17.84% and a γ*_a_* of 58.25%. For polyurea composites using the atmospheric pressure process, γ*_p_* values first decline and then rise with the increase in the SiO_2_ aerogel concentration, while the γ*_a_* values first go up and then decline. PA5N has the best mitigation performance, with a γ*_p_* of 12.66% and a γ*_a_* of 52.28%.
2. In thermal conductivity experiments, it is found that the addition of SiO_2_ aerogel is conducive to improving the thermal insulation performance of the materials, and the thermal insulation performance of all SiO_2_-aerogel-modified polyurea composites is better than that of PU. For polyurea composites with the vacuum process, the thermal insulation performance first goes up and then declines with the increasing concentration of SiO_2_ aerogel. PA3V has the best thermal insulation performance, with a thermal conductivity of 0.19621 W/m·K, which is 24.8% lower than PU. For polyurea composites with the atmospheric pressure process, the thermal insulation performance is significantly improved and slightly decreases (from 0.14174 W/m·K to 0.16793 W/m·K) with the increase in the SiO_2_ aerogel concentration. PA2N has the best thermal insulation performance, with a thermal conductivity of 0.14174 W/m·K, which is 45.65% lower than PU. These results indicate that the atmospheric pressure process is more conducive to improving the thermal insulation performance of polyurea composites.
3. In the LOI experiments, it is found that the vacuum and atmospheric pressure processes significantly affect the flame retardancy of the materials. For polyurea composites using the vacuum process, their LOI values are higher than PU. Among them, the LOI of PA7V reached 22.5%, indicating a certain degree of self-extinguishing performance. This shows that the SiO_2_ aerogel is conducive to improving the flame retardancy of polyurea composites when the vacuum process used. For polyurea composites using the atmospheric pressure process, all their LOI values are below 21%. Only LOI values of PA4N and PA5N are slightly higher than that of PU, which are 19.7% and 20.4%, respectively, indicating that the atmospheric pressure process is not conducive to improving the flame retardancy of polyurea.
4. In the thermogravimetric experiments, the thermal stability of polyurea composites is evaluated. For polyurea composites using the vacuum process, as the concentration of the SiO_2_ aerogel increases, the residual mass values increase (from 9.36% to 13.87%), the peak degradation temperatures increase (from 428.6 °C to 431.4 °C), and the peak degradation rates decline (from −20.74%/min to −18.82%/min). The experimental results show that the addition of the SiO_2_ aerogel is beneficial for improving the thermal stability of the polyurea composites when using the vacuum process. For polyurea composites using the atmospheric pressure process, the addition of the SiO_2_ aerogel can reduce the thermal stability of the polyurea composites. With the increase in the SiO_2_ aerogel concentration, the residual mass values (from 8.18% to 10.78%) and peak degradation rates (from −19.93%/min to −20.99%/min) go up. The results also show that the atmospheric pressure process damages the thermal stability of the polyurea composites compared with the vacuum process.

## Figures and Tables

**Table 1:** SiO_2_ aerogel mass concentration and process of modified polyurea composites.

Material	SiO_2_ Aerogel (Mass Concentration)	Process of Step2 and Step3
PA1V	1%	Vacuum (6 × 10^−2^ Pa)
PA2V	2%	
PA3V	3%	
PA5V	5%	
PA7V	7%	
PA10V	10%	
PA1N	1%	Atmospheric pressure (1.01 × 10^5^ Pa)
PA2N	2%	
PA3N	3%	
PA4N	4%	
PA5N	5%	

**Table 2:** LOI and thermal conductivity of polyurea with different filler concentration and process.

Material	Thermal Conductivity Factor (W/m·K)	Limiting Oxygen Index (%)
PU	0.26081	19.6
PA1V	0.25267	20.2
PA2V	—	21.2
PA3V	0.19621	21.2
PA5V	0.20616	21.4
PA7V	0.23566	22.5
PA10V	0.23648	20.1
PA1N	—	19.2
PA2N	0.14174	19.2
PA3N	0.14346	19.5
PA4N	0.15206	19.7
PA5N	0.16793	20.4

## Data Availability

The data generated or analyzed during this study are included in this published article. For further inquiries, please contact the corresponding author.
